# Different types of cartilage neotissue fabricated from collagen hydrogels and mesenchymal stromal cells via *SOX9*, *TGFB1* or *BMP2* gene transfer

**DOI:** 10.1371/journal.pone.0237479

**Published:** 2020-08-13

**Authors:** Manuel Weißenberger, Manuela H. Weißenberger, Mike Wagenbrenner, Tizian Heinz, Jenny Reboredo, Boris M. Holzapfel, Maximilian Rudert, Jürgen Groll, Christopher H. Evans, Andre F. Steinert

**Affiliations:** 1 Department of Orthopaedic Surgery, König-Ludwig-Haus, Orthopaedic Center for Musculoskeletal Research (OCMR), Julius-Maximilians-University Würzburg, Würzburg, Germany; 2 Department of Tissue Engineering and Regenerative Medicine, Julius-Maximilians-University Würzburg, Würzburg, Germany; 3 Department of Functional Materials in Medicine and Dentistry, Julius-Maximilians-University Würzburg, Würzburg, Germany; 4 Department of Physical Medicine and Rehabilitation, Musculoskeletal Gene Therapy Research Laboratory, Mayo Clinic, Rochester, MN, United States of America; Università degli Studi della Campania, ITALY

## Abstract

**Objective:**

As native cartilage consists of different phenotypical zones, this study aims to fabricate different types of neocartilage constructs from collagen hydrogels and human mesenchymal stromal cells (MSCs) genetically modified to express different chondrogenic factors.

**Design:**

Human MSCs derived from bone-marrow of osteoarthritis (OA) hips were genetically modified using adenoviral vectors encoding sex-determining region Y-type high-mobility-group-box (*SOX*) *9*, *transforming growth factor beta (TGFB) 1* or bone morphogenetic protein (*BMP) 2* cDNA, placed in type I collagen hydrogels and maintained in serum-free chondrogenic media for three weeks. Control constructs contained unmodified MSCs or MSCs expressing GFP. The respective constructs were analyzed histologically, immunohistochemically, biochemically, and by qRT-PCR for chondrogenesis and hypertrophy.

**Results:**

Chondrogenesis in MSCs was consistently and strongly induced in collagen I hydrogels by the transgenes *SOX9*, *TGFB1* and *BMP2* as evidenced by positive staining for proteoglycans, chondroitin-4-sulfate (CS4) and collagen (COL) type II, increased levels of glycosaminoglycan (GAG) synthesis, and expression of mRNAs associated with chondrogenesis. The control groups were entirely non-chondrogenic. The levels of hypertrophy, as judged by expression of alkaline phosphatase (ALP) and COL X on both the protein and mRNA levels revealed different stages of hypertrophy within the chondrogenic groups (*BMP2*>*TGFB1*>*SOX9*).

**Conclusions:**

Different types of neocartilage with varying levels of hypertrophy could be generated from human MSCs in collagen hydrogels by transfer of genes encoding the chondrogenic factors *SOX9*, *TGFB1* and *BMP2*. This technology may be harnessed for regeneration of specific zones of native cartilage upon damage.

## Introduction

Autologous chondrocyte transplantation (ACT) currently represents the standard of care for treating large, full-size chondral and osteochondral defects [1]. However, the application of ACT is limited, as it leads to donor site morbidity and yields only moderate cell numbers with limited differentiation capacites [2]. This is why mesenchymal stromal cells (MSCs) have appeared as a promising alternative for cell-based cartilage repair [3]. MSCs can be isolated from a variety of easily accessible tissues in high numbers and possess differentiation versatility, with bone-marrow being the most intensely investigated tissue source [4].

To develop a clinical application of MSC-based approaches for cartilage regeneration, a combination of MSCs with a beneficial 3D-microenvironment is necessary, containing an appropriate combination of biomaterials with differentiation factors [5]. Various biomaterials studied for this purpose comprise synthetic or natural polymers which prevent the loss of transplanted cells and simulate the natural structure of articular cartilage to promote chondrogenesis [5]. Among these, hydrogels retain large amounts of water, thereby imitating the highly hydrated extracellular matrix (ECM) in hyaline cartilage [6, 7]. Although natural hydrogels, like those formed by collagen fibers, lack stability, they include the benefits of high biocompatibility and biodegradability [6, 8, 9]. Especially collagen type I hydrogels are gaining popularity as scaffolds in cell-based cartilage repair due to their ready availability and safe use *in vivo* including several clinical trials [10–12].

Differentiation of MSCs toward a chondrogenic phenotype also requires specific stimulation which can include a great number of variables such as hypoxia, mechanical loading or the delivery of chondrogenic growth factors [9]. In this context, relatively little attention has been dedicated to the zonal organization of neocartilage tissue and how it depends upon the specific differentiation factor that is used. The most well studied soluble factors for chondrogenic induction of MSCs are the members of the transforming growth factor (TGF)-β family, such as TGF-β1 (encoded by *TGFB1*) or bone morphogenetic protein (BMP)-2 (encoded by *BMP2*). Although, chondrogenic induction could be achieved using these factors under certain chondrogenic conditions *in vitro* [13], the problems with this approach include adequate delivery of these factors at sufficiently high and sustained concentrations *in vivo* and the high levels of hypertrophic chondrocytes which produce a matrix rich in type X collagen and express osteogenic marker genes.

To address issues surrounding the delivery and choice of chondrogenic factors, we [14, 15] and others [16, 17] have developed new approaches for delivering growth factors to MSCs to induce chondrogenic differentiation. In particular, the combination of gene transfer and biomaterials for cartilage tissue engineering has shown promising results (Table 1). In previous work using adenoviral gene transfer to MSCs we and others could show that marrow-derived MSCs were highly amenable to adenoviral transduction of *TGFB1* and *BMP2* resulting in strong chondrogenic induction in pellet cultures *in vitro* [15] and cartilage defects *in vivo* [26]. However, the issue of chondrogenic hypertrophy *in vitro* and subsequent osteogenic induction within cartilage defects *in vivo* remains a problem.

**Table 1:** Overview of studies focusing on the combination of gene transfer and biomaterials for cartilage tissue engineering.

*Study*	*Cells*	*Growth factors/signaling pathways*	*Biomaterial*	*Observation*
Noth et al. [13]	BMSCs	Soluble growth factors TGF-ß1, BMP-2 or TGF-ß1 + BMP-2	Collagen I hydrogel	Successful chondrogenesis in vitro
Li et al. [18]	BMSCs	Plasmid based gene transfer of TGF-ß1	PGA/fibrin hydrogel	Repair of full thickness cartilage defects in vivo in rabbit models
Xia et al. [19]	BMSCs	Adenoviral gene transfer of TGF-ß1	PGA scaffold	Successful chondrogenesis in vivo in mice models
Steinert et al. [15]	BMSCs	Adenoviral gene transfer of different combinations of TGF-ß1, BMP-2 and IGF-1	Pellet cell culture	Successful chondrogenesis in vitro
Wang et al. [20]	BMSCs	Adenoviral gene transfer of TGF-ß3 and BMP-2	Demineralized bone matrix	Repair of full thickness cartilage defects in vivo in pig models
Leng at al. [21]	BMSCs	Plasmid based gene transfer of IGF-1	Calcium Alginate hydrogel	Repair of full thickness cartilage defects in rabbit models
Cao et al. [22]	BMSCs	Adenoviral gene transfer of SOX-9	PGA scaffold	Successful chondrogenesis in vitro, repair of full thickness cartilage defects in vivo in rabbit models
Venkatesan et al. [23]	BMSCs	Recombinant adeno-associated viral gene transfer of SOX-9	Pellet cell culture	Successful chondrogenesis in vitro
Venkatesan et al. [24]	BMSCs	Recombinant adeno-associated viral gene transfer of SOX-9	Fibrin/polyurethane scaffold	Successful chondrogenesis in vitro under hydrodynamic culture conditions
Lu et al. [25]	ADSCs	Baculoviral gene transfer of TGF-ß3 and BMP-6	PLGA scaffold	Repair of full thickness cartilage defects in vivo in rat models
Lee and Im [26]	ADSCs	Retroviral gene transfer of SOX-5, SOX-6 and SOX-9	Fibrin hydrogel	Repair of full thickness cartilage defects in vivo in rat models
Goodrich et al. [27]	AC	Adenoviral gene transfer of IGF-1	Fibrinogen hydrogel	Repair of osteochondral defects in vivo in equine models
Kaul et al. [28]	AC	Plasmid based gene transfer of FGF-2	Alginate hydrogel	Repair of osteochondral defects in vivo in rabbit models
Orth et al. [29]	AC	Plasmid based gene transfer of IGF-1 and FGF-2	Alginate hydrogel	Repair of osteochondral defects in vivo in rabbit models

Adipose stem cells (ADSCs), articular chondrocytes (AC), bone marrow-derived mesenchymal stromal cells (BMSCs), bone morphogenetic protein (BMP), fibroblast growth factor (FGF), insulin like growth factor (IGF), polyglycolic acid (PGA), polylactide-co-glycolic acid (PLGA), sex-determining region Y-box 9 (SOX-9), transforming growth factor (TGF).

Recently, the transcription factor sex-determining region Y-type high-mobility-group-box (SOX) 9 (encoded by *SOX9*) has been employed for chondrogenic induction of MSCs via gene delivery using adeno-associated virus (AAV) [16] or adenoviral vectors [30], which elicited significantly reduced levels of hypertrophy in pellet cultures *in vitro*. Because SOX9 is an intracellular protein, delivery by means other than gene transfer is difficult. The purpose of our current study was to combine the gene transfer of chondrogenic factors such as *TGFB1*, *BMP2* and *SOX9* with a method of cell culture and delivery that has potential for *in vivo* application. Therefore, we used type I collagen hydrogels that are in clinical use rather than pellet culture and hypothesized that we could fabricate neo-cartilaginous tissues at different stages of cartilage hypertrophy depending upon the specific gene product encoded by the transferred cDNAs.

## Materials and methods

### Generation and propagation of recombinant adenoviral vectors

Adenoviral vectors carrying green fluorescent protein (*GFP*) from jellyfish, the transcription factor *SOX9* with a *GFP* fusion construct, *TGFB1* and *BMP2* cDNA were generated by *cre-lox* recombination, amplified, purified and used as described before [31–33]. The resulting vectors were termed Ad.*GFP*, Ad.*SOX9*, Ad.*TGFB1* and Ad.*BMP2*. The preparation of recombinant adenovirus suspensions was carried out by amplification in 293 cells followed by purification using three consecutive CsCl gradients. Virus stock titers were estimated by optical density at 260 nm and standard plaque assay, and ranged between 1012–10^13^ particles/mL.

### Culture and transduction of human marrow-derived MSCs

After approval of the institutional review board of the Julius-Maximilians-University Würzburg and informed consent of all patients, MSCs were isolated from the femoral head of five patients undergoing total hip arthroplasty due to primary osteoarthritis using a protocol described earlier [33, 34]. Briefly, the collected cells were spun, re-suspended and plated at 2–3 x 10^8^ nucleated cells per 150 cm^2^ flask (Falcon, Beckton Dickinson Labware, Franklin Lakes, NJ) using complete DME/F-12 medium (DMEM) containing 10% FBS and 1% penicillin/streptomycin, 1 ng/mL FGF-2 (all Invitrogen GmbH, Darmstadt, Germany). After 3 days, unattached cells were removed, and adherent cells were cultured at 37°C in a humidified atmosphere of 95% air and 5% CO_2_ in DMEM. Medium changes were performed every 3–4 days. As soon as cells reached confluency (approximately 1.2 x 10^6^ cells/150 cm^2^ flask), the cultures were washed with phosphate buffered saline (PBS) and infected in 750 μL serum-free DMEM for 2 hours at a dose of 5 x 10^2^ infectious particles/cell of Ad.*GFP* (control group), Ad.*SOX9*, Ad.*TGFB1* or Ad.*BMP2*, or remained uninfected. The supernatant was removed after 2 hours of viral infection and replaced with DMEM.

### Fabrication and culture of collagen hydrogel constructs

The type I collagen hydrogel was derived from collagen fibers isolated from rat tails (8–10 weeks old, both sexes) as described previously [13]. After washing the collagen fibres with PBS and sterilizing them with 70% EtOH, they were dissolved in acetic acid (0.1%) at a concentration of 6 mg/mL. To obtain a collagen matrix, the gel was mixed with a gel neutralization solution (GNL), that contained DMEM, FCS, and HEPES buffer. Following neutralization, 3 x 10^5^ cells were suspended in 0.15 mL GNL and transduced at 5 x 10^2^ infectious particles (ip)/cell for each vector with non-transduced or marker gene (*GFP*) transduced cultures as controls. Thereafter, 0.15 mL type I collagen hydrogel was added and mixed carefully in order to avoid air inclusion. Then 1 mL of the mixture was pipetted into a polypropylene, v-bottom 96-well plate (Corning, Corning, NY, USA). Gel polymerization was allowed in a 5% humidified atmosphere at 37°C for 30 min. Then 0.1 mL of a defined cell culture medium (serum-free DMEM containing 1 mM pyruvate, 1% ITS + Premix, 37.5 mg/ml ascorbate-2-phosphate and 10^−7^ M dexamethasone (all Sigma, St. Louis, MO, USA) was added to each well for overnight incubation. The next day the gels were put in a 24-well plate (Corning, Corning, NY, USA) and cultured with 2 mL medium which was changed every 2–3 days until harvesting.

### Assessment of transgene expression

Transgene expression for the marker gene *GFP* and the chondrogenic factor *SOX9*, which is fused to *GFP* in this construct, was measured by fluorescence microscopy, whereas *TGFB1* and *BMP2* expression were measured with commercially available ELISA kits as directed by the supplier (R&D Systems) and 24 hour-conditioned media collected at days 3, 7, 14 and 21.

### Biochemical assays

For assessment of cell proliferation, glycosaminoglycan (GAG) synthesis and alkaline phosphatase (ALP; encoded by *ALPL*) activity biochemical assays were performed as described earlier [33]. Briefly, for detection of cell proliferation a quantitative adenosine 5´-triphosphate (ATP) assay was performed using the CellTiter-Glo^®^ Luminescent Cell Viability Assay (Promega). After papain digestion (1 μg/mL, Sigma), synthesis of GAG was evaluated by reaction with 1,9-dimethylmethylene blue (DMMB) using the Blyscan^TM^ Sulfated Glycosaminoglycan Assay (Biocolor Ltd., Newtownabbey, Northern Ireland). For detection of ALP activity, a kit (Sigma) was used that measured ALP activity based upon the conversion of p-nitrophenyl phosphate to p-nitrophenol and inorganic phosphate; absorbance at 405 nm was measured in an ELISA plate reader. Using the Quant-iT™ PicoGreen® kit (Invitrogen), the GAG content and the ALP activity were normalized to DNA content.

### Histology and immunohistochemistry

Type I collagen hydrogel aggregates were fixed at day 21 in 4% paraformaldehyde, followed by dehydration, paraffin embedding, sectioning and staining with hematoxylin/eosin (H&E), alcian blue and ALP (all Sigma) according to previously published protocols [33]. Alternate sections were used for immunohistochemistry. The following antibody treatments were used: type II collagen (COL II) (pepsin (1 mg/ml; Sigma)/monoclonal anti-COL II antibodies (Acris Antibodies GmbH, Hiddenhausen, Germany)); chondroitin-4-sulfate (CS4) (chondroitinase ABC (5 U/mL; Sigma)/monoclonal anti-CS4 antibodies (Millipore GmbH, Schwalbach, Germany)); type X collagen (COL X) (0.25% trypsin; Sigma)/polyclonal anti-COL X antibodies (Calbiochem, Bad Soden, Germany)). Visualization of the immunostainings was performed by treatment with Advance™ HRP link and Advance™ HRP enzyme (Dako, Hamburg, Germany) followed by diaminobenzidine staining (DAB kit; Sigma). Thereafter, the slides were counterstained with hemalaun (Merck, Darmstadt, Germany). Controls with non-immune IgG (Sigma) were performed for all immunohistochemical analyzes.

### RNA extraction and reverse-transcription quantitative PCR analyzes

RNA was extracted from the hydrogel constructs at days 3, 7, 14 and 21, and 2 μg RNA from each group used for reverse transcription using random hexamer primers (Table 2) and BioScript reverse transcriptase (Bioline GmbH, Luckenwalde, Germany). Quantitative PCR (qPCR) was performed in triplicate with 1 μL cDNA, 10 μL KAPA SYBR FAST Universal 2x qPCR Master Mix (peqlab Biotechnologie GmbH) and 1 μL of gene specific primers. Eventually, qPCR was performed with Opticon DNA Engine (MJ Research, Waltham, USA) under the following conditions: 95°C for 3 min; 40 cycles: 95°C for 15 s; 58°C for 20 s; 72°C for 30 s; thereafter the melting curve was analyzed. Final results were calculated using the Δ Δ-C_T_ method.

**Table 2:** Primer sequences and product sizes, for quantitative RT-PCR.

*Gene*	*RT-PCR primer sequences (5’-3’)*	*Annealing temp*. *(°C)*	*Product size (bp)*	*Cycles*
**Chondrogenic markers**				
*COL2A1*	Sense: TTTCCCAGGTCAAGATGGTC	58	374	35
	Antisense: CTTCAGCACCTGTC CACCA			
*SOX9*	Sense: ATCTGAAGAAGGAGAGCGAG	58	263	35
	Antisense: TCAGAAGTCTCCAGAGCTTG			
**Hypertrophy and osteogenic markers**				
*COL10A1*	Sense: CCCTTTTTGCTGCTAGTATCC	54	468	25
	Antisense: CTGTTGTCCAGGTTTTCCTGGCAC			
*ALPL*	Sense: TGGAGCTTCAGAAGCTCAACACCA	51	454	35
	Antisense: TCTCGTTGTCTGAGTACCAGTCC			
**Internal control**				
*EF-1α*	Sense: AGGTGATTATCCTGAACCATCC	54	234	25
	Antisense: AAAGGTGGATAGTCTGAGAAGC			

### Statistical analyzes

Numeric data from the ELISA, ATP, GAG, DNA, and ALP analyzes were expressed as mean values, range and standard deviation (SD), with each experiment being performed in triplicate or quadruplicate (n = 3–4), and repeated on at least 3–5 individual marrow preparations from different patients (n = 3–5), as indicated in the respective experiments. Numeric data were subjected to variance analysis (one or two factor ANOVA) and statistical significance between the different groups and timepoints was determined by the Friedman-Test and the Kruskal Wallis-Test with p < 0.05 being considered significant.

## Results

### Transgene expression by genetically-modified MSCs in collagen hydrogel constructs

**Fig 1:**
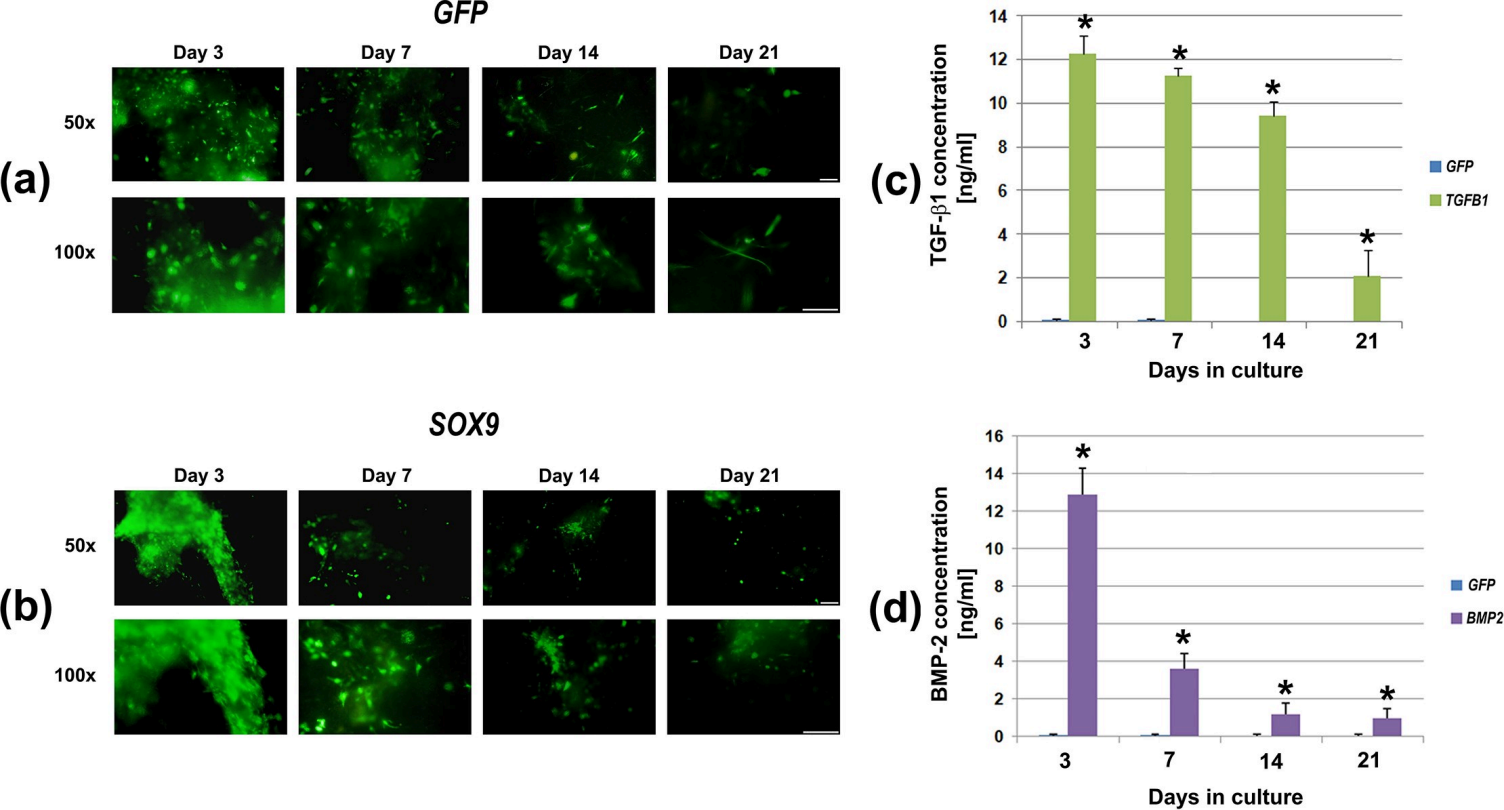
Transgene expression by MSCs in hydrogels during 21 days of culture following adenoviral gene transfer of *GFP*, *SOX9*, *TGFB1* or *BMP2*. Primary MSCs were transduced with Ad.*GFP*, Ad.*SOX9*, Ad.*TGFB1* or Ad.*BMP2* at 5 x 10^2^ vp/cell for each vector, seeded into type I collagen hydrogels and analyzed for the respective transgene expression during 21 days. Expression of the *GFP* transgene was analyzed by fluorescence microscopy in the hydrogels modified with Ad.*GFP* (a) or Ad.*SOX9/GFP* (b). Expression of the *TGFB1* (c) and *BMP-2* (d) transgenes was measured by ELISA. The data represent mean values ± SD from measurements of supernatants of n = 3 hydrogel aggregates per condition and time point; m = 3 marrow preparations were analyzed. Each experiment was performed in triplicate. Statistically different values from GFP+ control hydrogels are marked by asterisks (p < 0.05).

MSCs modified with adenoviral vectors encoding *GFP* (control), *SOX9*, *TGFB1* or *BMP2* and contained in type I collagen hydrogel constructs expressed high levels of the respective transgenes at day 3 of culture. The transgene expression in the negative control group *GFP* and transgene group S*OX9* measured by fluorescence microscopy showed high levels at day 3, decreasing progressively at later time points (Fig 1A and 1B). Concentrations of 11 to 13 ng/mL of TGF-**β**1 (Fig 1C) or 12 to14 ng/mL BMP-2 (Fig 1D) were measured, with declining values during the three weeks time-course. In the marker gene controls, the amount of each growth factor transgene was persistently low (< 0,01 ng/mL).

### Histological and immunohistochemical analyzes of chondrogenic differentiation

**Fig 2:**
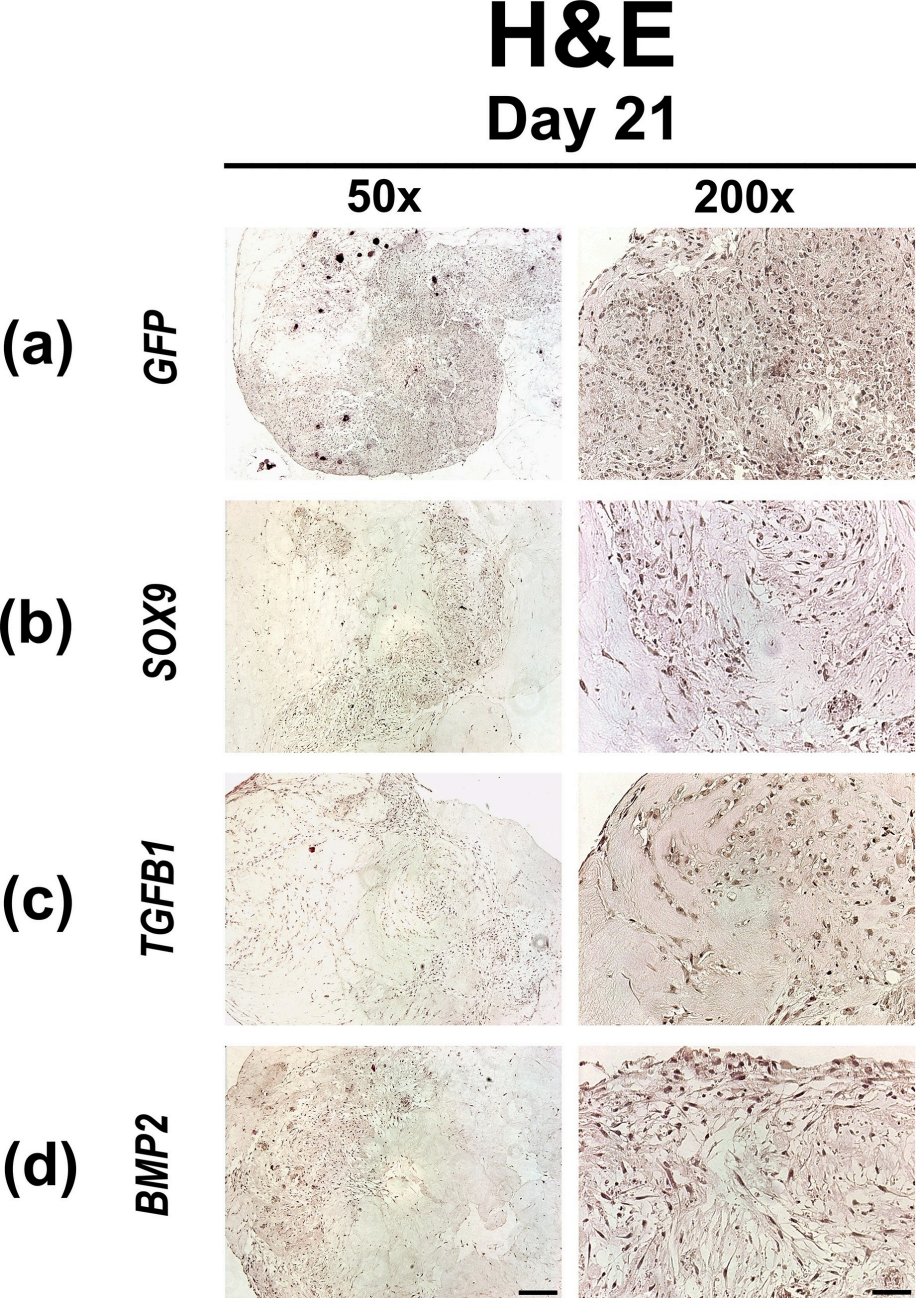
Histological appearance of MSC hydrogels after chondrogenic differentiation by adenoviral gene transfer of *GFP*, *SOX9*, *TGFB1* or *BMP2*. MSCs in monolayer cultures were transduced with Ad.*GFP* (Control; a), Ad.*SOX9* (b), Ad.*TGFB1* (c) or Ad.*BMP2* (d) at 5 x 10^2^ vp/cell for each vector as indicated, seeded into hydrogel aggregates 24 h post infection and cultured in serum-free medium for 21 days. Representative examples after 21 days are shown, that were stained with H & E for assessment of cell morphology and cellularity. (a-d) Panels are shown at low (50x; bar = 200 μm) or high (200x; bar = 50 μm) magnification as indicated.

**Fig 3:**
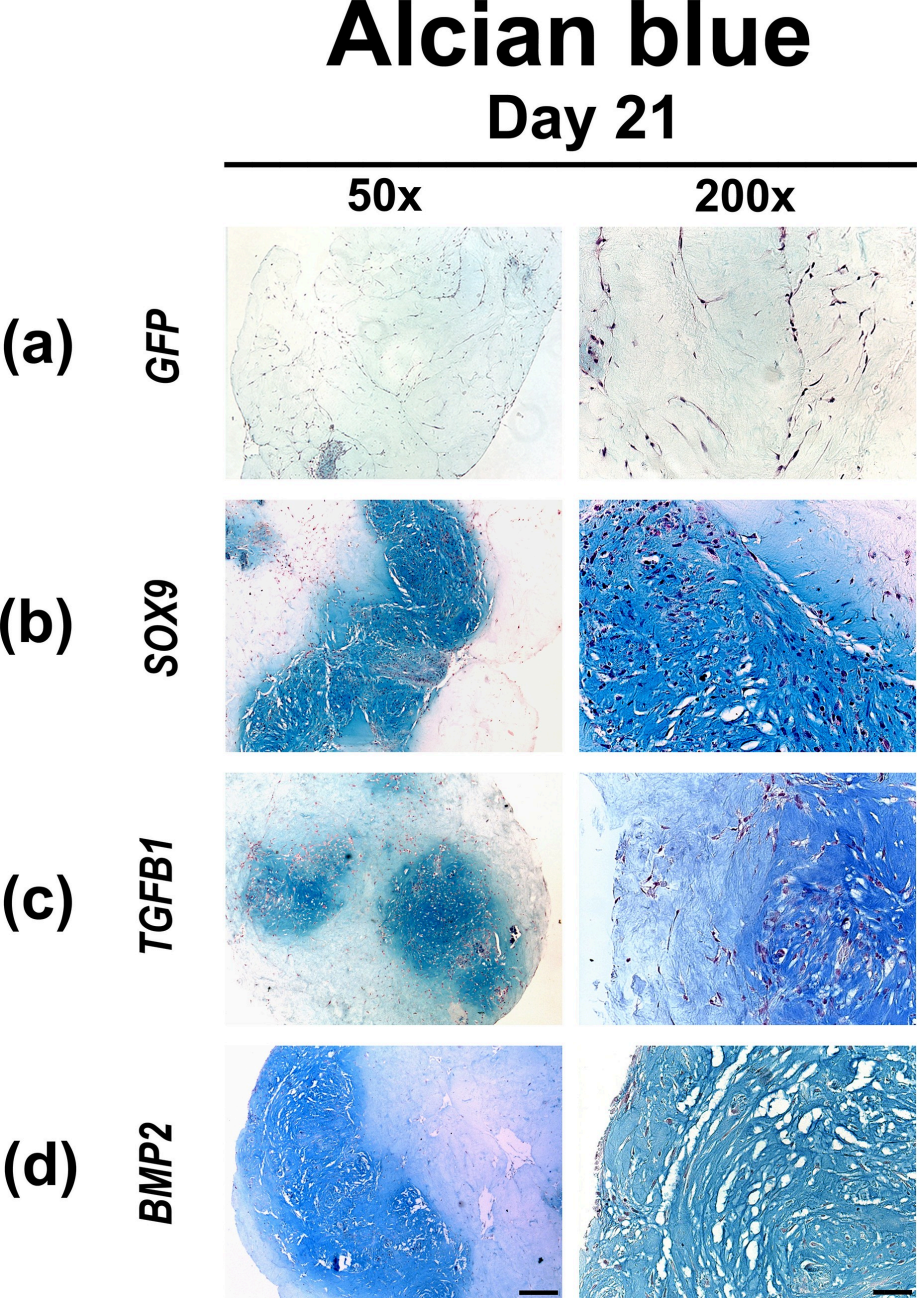
Histological appearance of MSC hydrogels after chondrogenic differentiation by adenoviral gene transfer of *SOX9*, *TGFB1* or *BMP2*. MSCs in monolayer cultures were infected with Ad.*GFP* (Control; a), Ad.*SOX9* (b), Ad.*TGFB1* (c) or Ad.*BMP2* (d) at 5 x 10^2^ vp/cell for each vector as indicated, seeded into hydrogel aggregates 24 h post infection and cultured in serum-free medium for 21 days. Representative examples after 21 days are shown, that were stained with alcian blue for evaluation of matrix proteoglycan content. (a-d) Panels are shown at low (50x; bar = 200 μm) or high (200x; bar = 50 μm) magnification as indicated.

The degree of cellularity of the respective hydrogels containing MSCs transduced with Ad.*GFP*, Ad.*SOX9*, Ad.*TGFB1* and Ad.*BMP2* after 21 days in culture is shown by H & E staining of representative hydrogel constructs in Fig 2. After 21 days of culture the Ad.*GFP* (Fig 2A) hydrogel constructs showed a higher degree of cellularity compared to Ad.*SOX9* (Fig 2B) and Ad.*BMP2* (Fig 2D) hydrogel constructs. The Ad.*TGFB1* (Fig 2C) hydrogels showed the lowest degree of cellularity with loose complexes of cells surrounded by vast amounts of extracellular matrix. Alcian blue staining (Fig 3) revealed strong metachromatic staining for proteoglycans in the extracellular matrix deposited by the Ad.*SOX9*, Ad.*TGFB1* and Ad.*BMP2* (Fig 3B–3D) transfected MSCs in type I collagen hydrogel constructs, in comparison to the Ad.*GFP* (Fig 3A) transfected control group.

**Fig 4:**
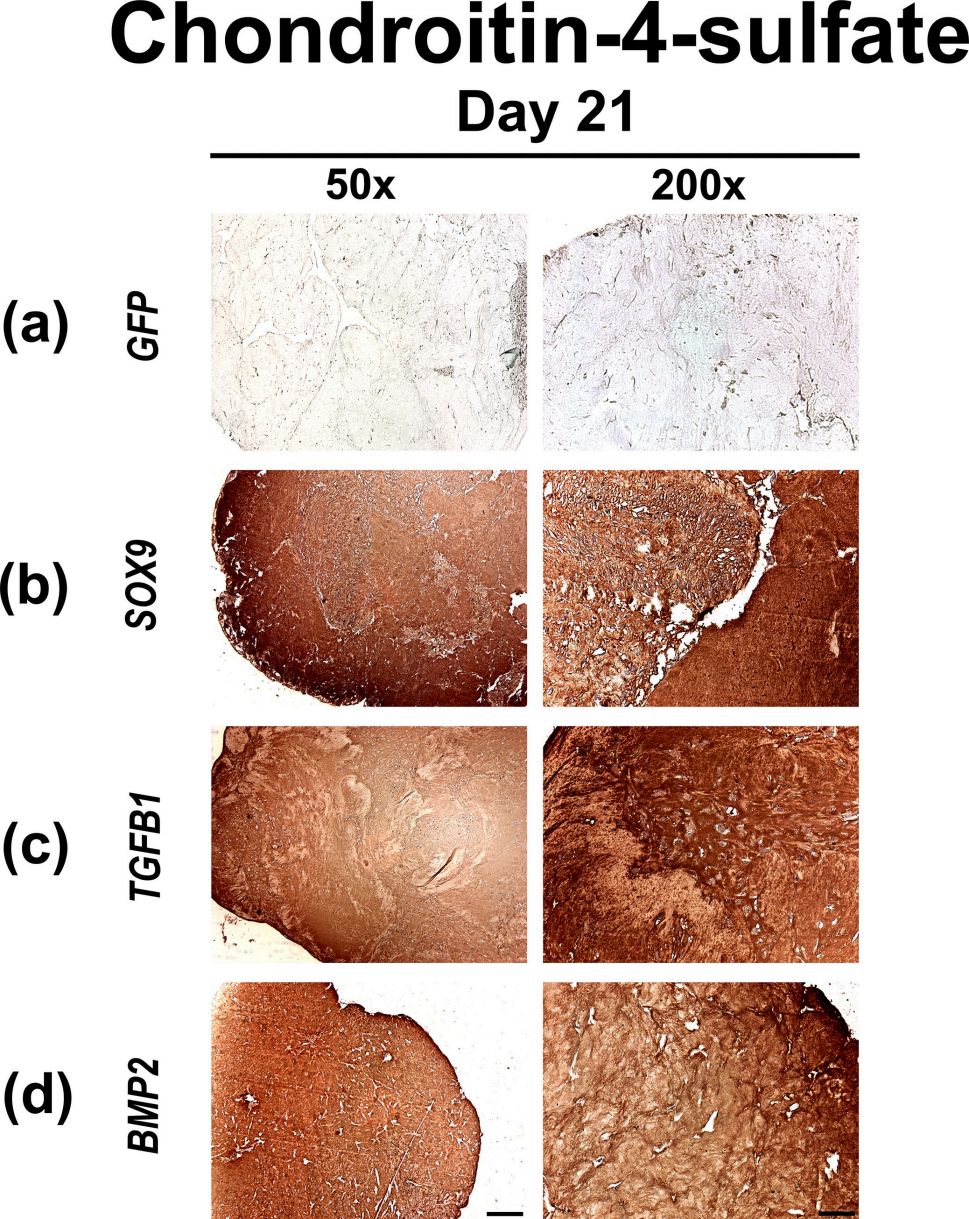
Immunohistochemical analyzes for chondroitin-4-sulfate in MSC hydrogels after chondrogenic differentiation by adenoviral gene transfer of *SOX9*, *TGFB1* or *BMP2*. Monolayer cultures of MSCs were infected with Ad.*GFP* (Control; a), Ad.*SOX9* (b), Ad.*TGFB1* (c) or Ad.*BMP2* (d) at 5 x 10^2^ vp/cell for each vector as indicated, seeded into hydrogel aggregates 24 h after infection and cultured in serum-free medium for 21 days. Immunohistochemical staining for chondroitin-4-sulfate (CS4), one of the proteoglycan matrix components, was performed on representative sections after 21 days. (a-d) Panels are reproduced at low (50x; bar  =  200 μm) or high (200x; bar  =  50 μm) magnification as indicated, and regions of positive immunostaining appear brown.

Accordingly, immunohistochemical stainings for CS4 (Fig 4), part of the polysaccharide fractions of cartilage GAGs, showed increased production by MSCs at day 21 of culture in the Ad.*SOX9*, Ad.*TGFB1* and Ad.*BMP2* (Fig 4B–4D) hydrogel constructs in comparison to the Ad.*GFP* controls (Fig 4A) which showed only very low levels of staining for CS4.

**Fig 5:**
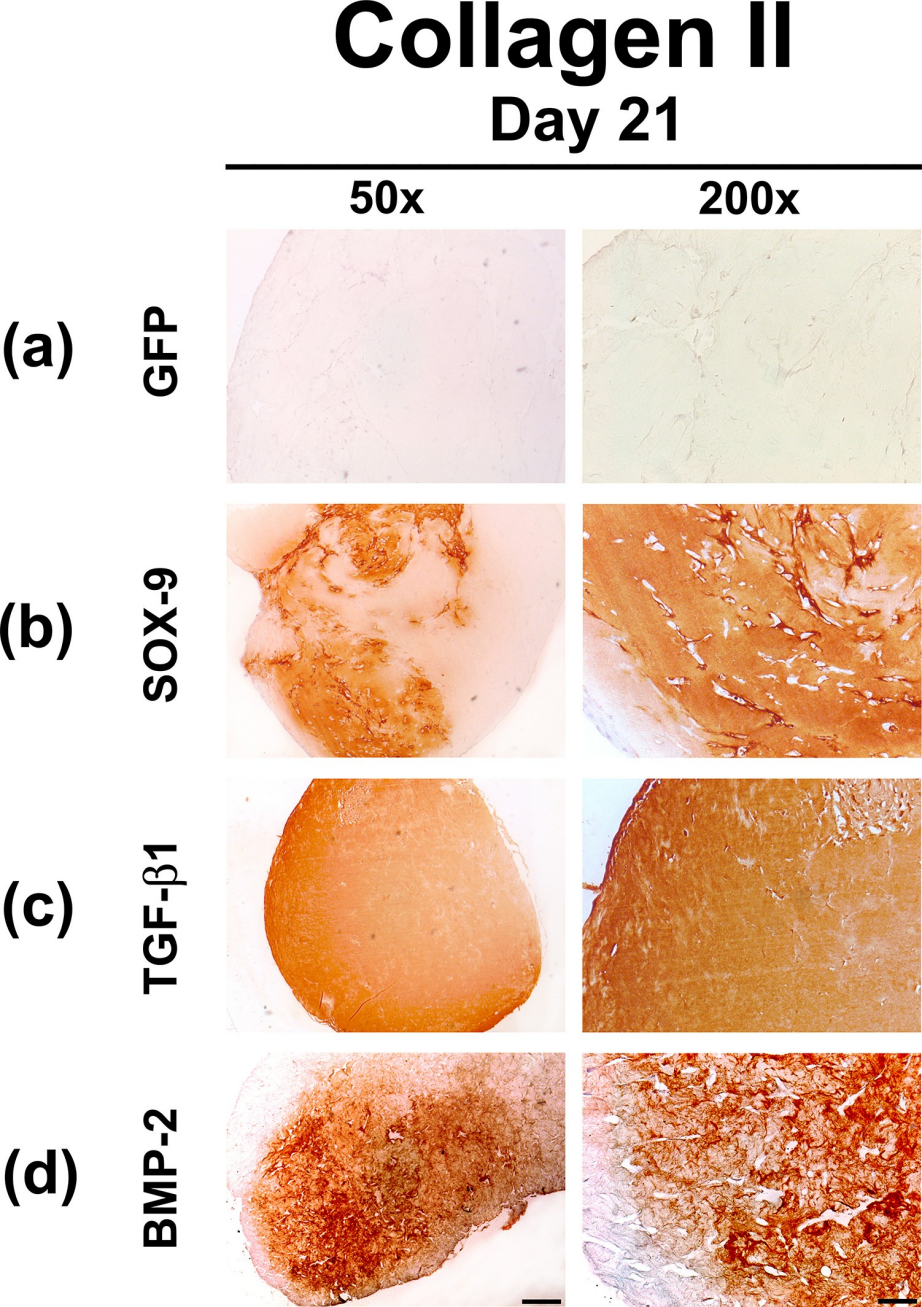
Immunohistochemical analyzes for type II collagen in MSC hydrogels after chondrogenic differentiation by adenoviral gene transfer of *SOX9*, *TGFB1* or *BMP2*. MSCs in monolayer cultures were infected with Ad.*GFP* (Control; a), Ad.*SOX9* (b), Ad.*TGFB1* (c) or Ad.*BMP2* (d) at 5 x 10^2^ vp/cell for each vector as indicated, seeded into hydrogel aggregates 24 h post infection and cultured in serum-free medium for 21 days. Immunohistochemical staining for type II collagen (COL II), the predominant collagen in hyaline cartilage, was performed on representative sections after 21 days. (a-d) Panels are shown at low (50x; bar = 200 μm) or high (200x; bar = 50 μm) magnification as indicated, and regions of positive immunostaining appear brown.

Similar results were observed in the immunohistochemical stainings for COL II (Fig 5), the main cartilage matrix protein, which proved increased production of the molecule by MSCs at day 21 of culture in the Ad.*SOX9*, Ad.*TGFB1* and Ad.*BMP2* (Fig 5B–5D) hydrogel constructs in comparison to the Ad.*GFP* (Fig 5A) controls which low levels of COL II staining. Distinct differences in the expression levels of all mentioned chondrogenic markers between the *SOX9*, *TGFB1* and *BMP2* transduced hydrogel constructs were not evident.

### Immunohistochemical analyzes of chondrogenic hypertrophy

**Fig 6:**
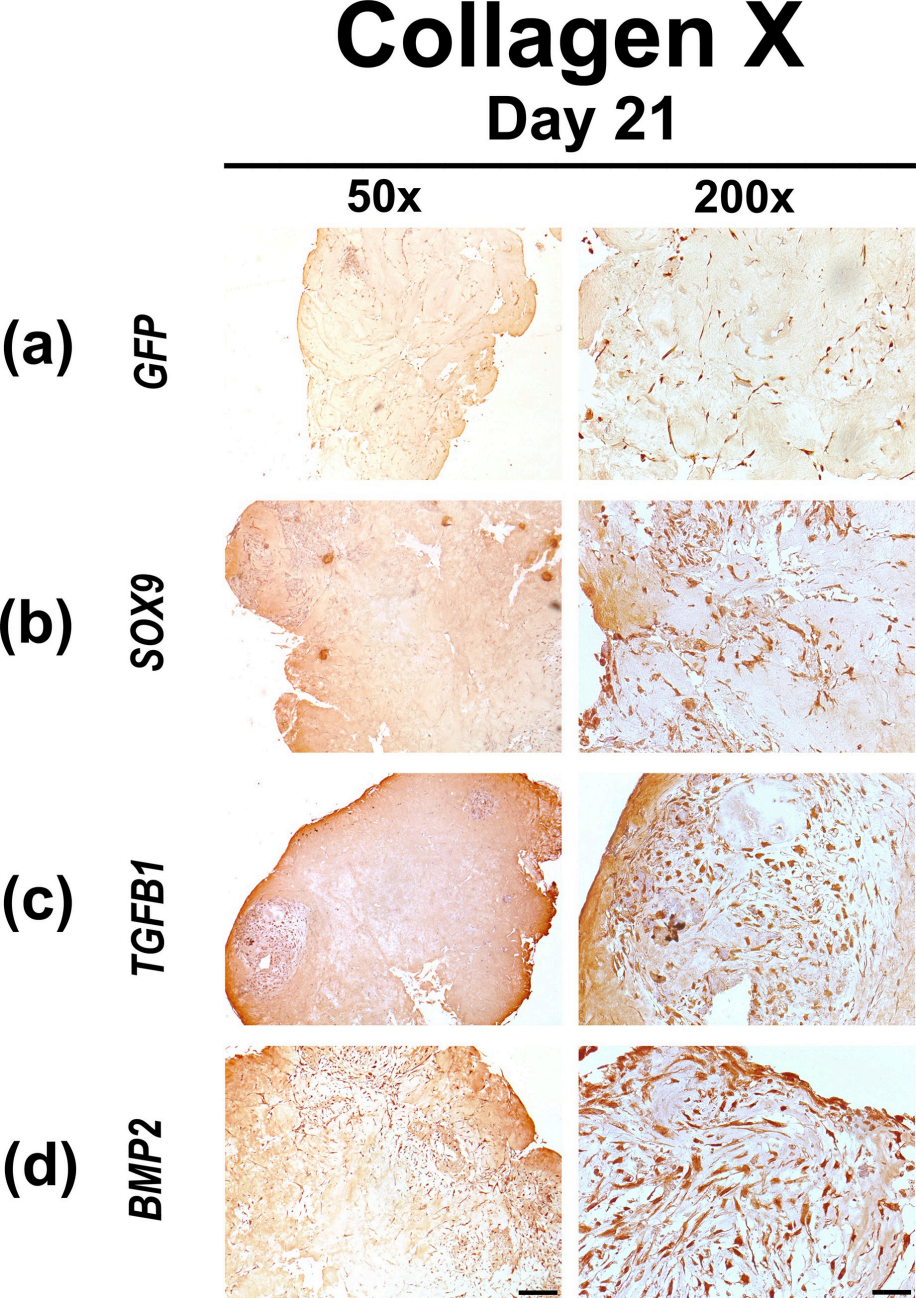
Immunohistochemical analyzes for type X collagen in MSC hydrogels after chondrogenic differentiation by adenoviral gene transfer of *SOX9*, *TGFB1* or *BMP2*. MSCs in monolayer cultures were infected with Ad.*GFP* (Control; a), Ad.*SOX9* (b), Ad.*TGFB1* (c) or Ad.*BMP2* (d) at 5 x 10^2^ vp/cell for each vector as indicated, seeded into hydrogel aggregates 24 h post infection and cultured in serum-free medium for 21 days. Immunohistochemical staining for type X collagen (COL X) was performed on representative sections after 21 days. (a-d) Panels are shown at low (50x; bar = 200 μm) or high (200x; bar = 50 μm) magnification as indicated; regions of positive immunostaining appear brown.

**Fig 7:**
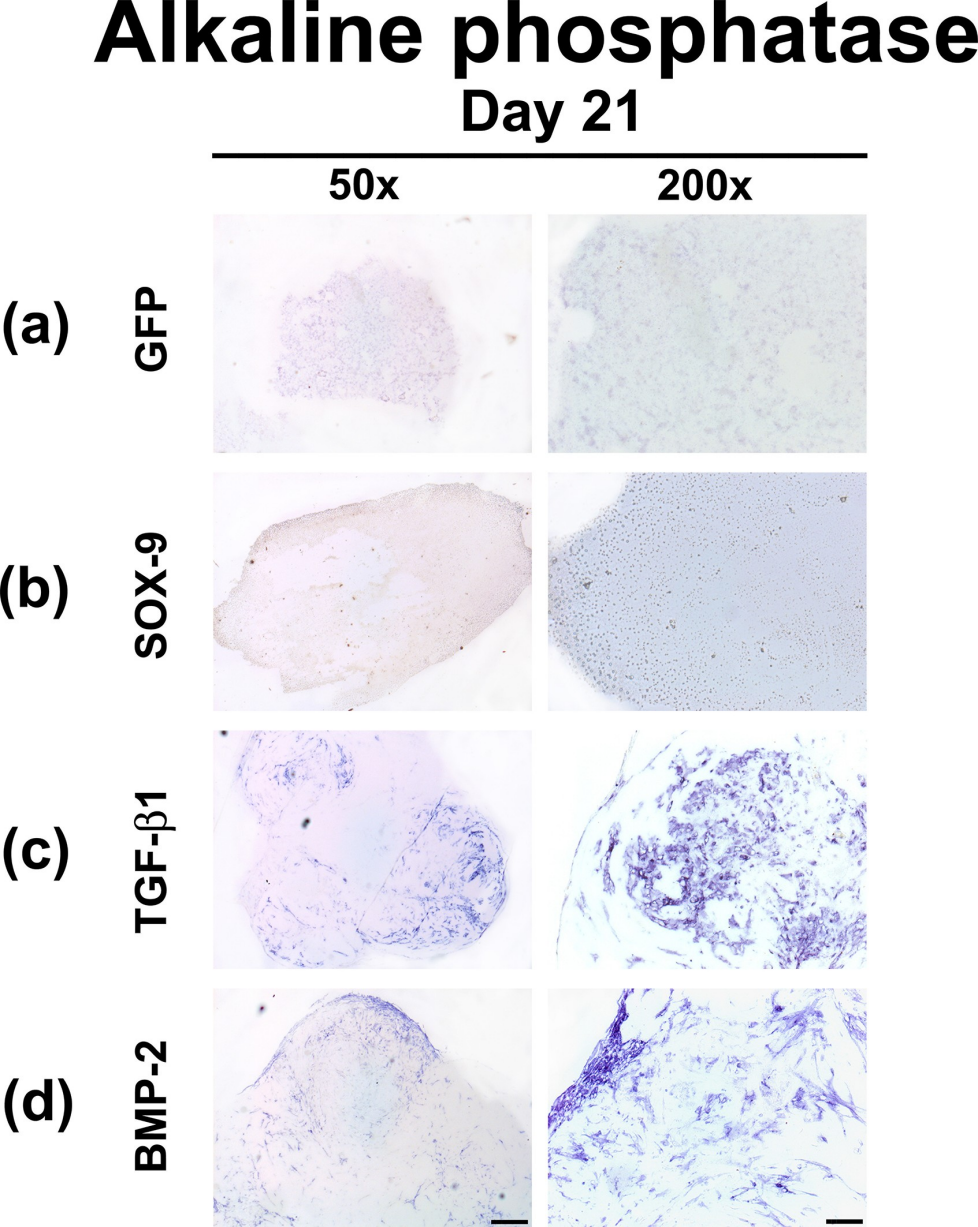
Histochemical analyzes for alkaline phosphatase in MSCs hydrogels after chondrogenic differentiation by adenoviral gene transfer of *SOX9*, *TGFB1* or *BMP2*. MSCs in monolayer cultures were infected with Ad.*GFP* (Control; a), Ad.*SOX9* (b), Ad.*TGFB1* (c) or Ad.*BMP2* (d) at 5 x 10^2^ vp/cell for each vector as indicated, seeded into hydrogel aggregates 24 hours post infection and cultured in serum-free medium for 21 days. Histochemical staining for alkaline phosphatase (ALP) was performed on representative sections after 21 days. (a-d) Panels are shown at low (50x; bar = 200 μm) or high (200x; bar = 50 μm) magnification as indicated, and regions of positive staining appear blue.

Chondrocyte hypertrophy was assessed by immunohistochemical analyzes for COL X (Fig 6) and ALP (Fig 7). The control group Ad.*GFP* showed only limited immunostaining of COL X at day 21 (Fig 6A). Similarly, only weak COL X staining could be seen in the hydrogel constructs containing chondrogenic differentiated MSCs transduced with Ad.*SOX9* (Fig 6B). In contrast, after 21 days intense COL X staining could be observed in the Ad.*TGFB1* and Ad.*BMP2* hydrogel constructs (Fig 6C and 6D).

Correspondingly, no or only very weak staining for ALP was detected in the type I collagen hydrogels containing MSCs transduced with Ad.*GFP* which served as a control group (Fig 7A), as well as in the hydrogels containing MSCs modified with Ad.*SOX9* (Fig 7B). In contrast, hydrogels containing MSCs that were transduced with Ad.*TGFB1* and Ad.*BMP2* showed increased staining for ALP, in particular in the extracellular matrix of the outer rim of the aggregates (Fig 7C and 7D).

### Biochemical assays for cell proliferation, DNA and GAG Content, and ALP activity

**Fig 8:**
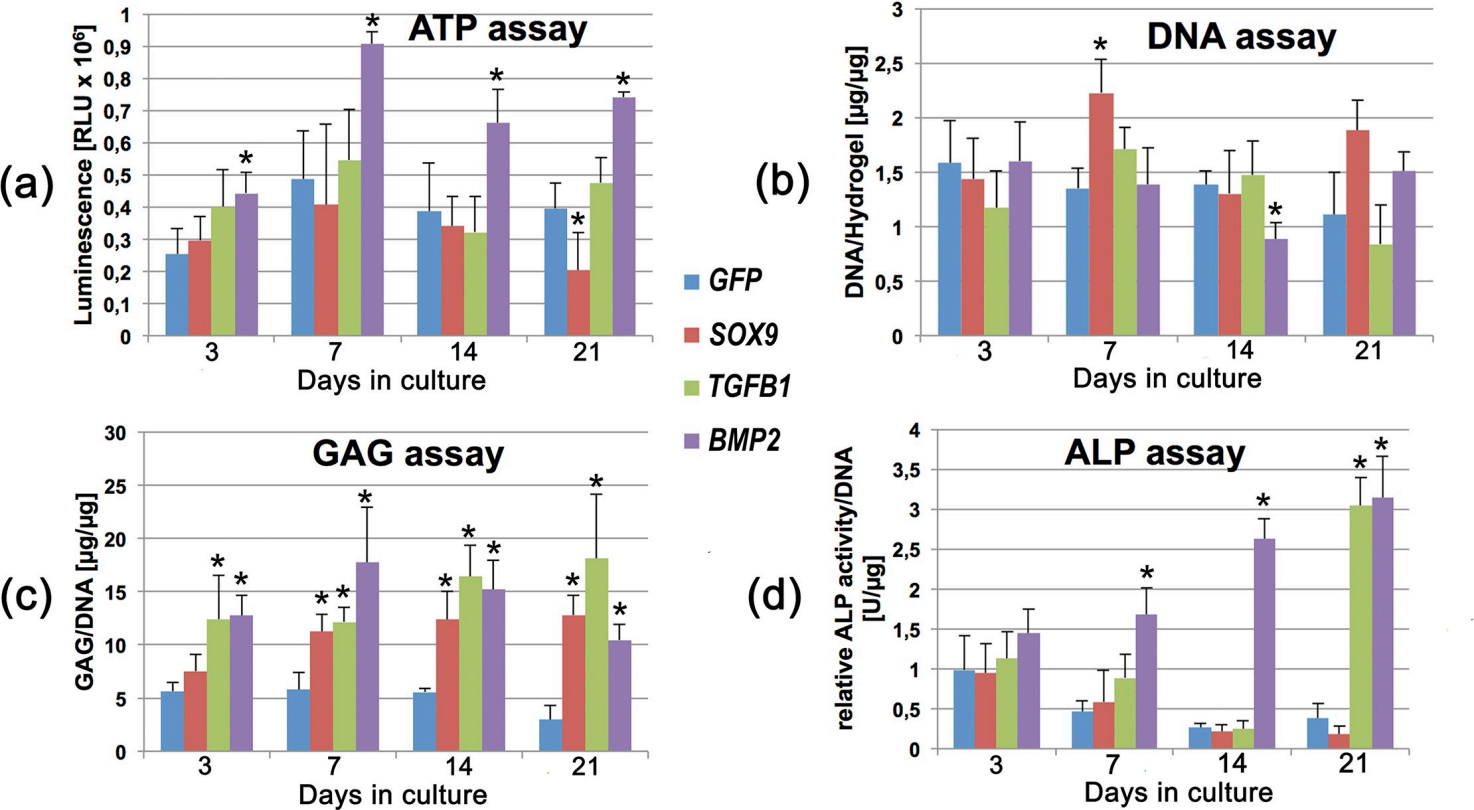
Biochemical composition of MSC hydrogels during three weeks of culture following adenoviral gene transfer of *SOX9*, *TGFB1* or *BMP2*. Primary MSCs in monolayer cultures were infected with Ad.*GFP* (Control; a), Ad.*SOX9*, Ad.*TGFB1* or Ad.*BMP2* at 5 x 10^2^ vp/cell for each vector as indicated, seeded into hydrogel aggregates 24 h post infection and cultured in serum-free medium for 21 days. (a) Cell proliferation was quantified by ATP assay at days 3, 7, 14 and 21. (b) The concentration of DNA was measured at days 3,7,14 and 21. (c) In addition, the GAG assay was performed for all *SOX9*, *TGFB1* and *BMP2* modified hydrogels. (d) As a quantitative parameter for hypertrophy ALP activity was measured and normalized to the DNA content. (a-d) The data represent mean values ± SD from n = 3 aggregates per condition and time point from marrow preparations of m = 3 different patients. Each experiment was performed in quadruplicate. Asterisks indicate values that are statistically different (p < 0.05) from marker gene vector–transduced control cultures.

After 21 days of culture in type I collagen hydrogels we performed ATP cell proliferation assays to estimate changes of cell viability in MSCs after adenoviral transduction with Ad.*GFP*, Ad.*SOX9*, Ad.*TGFB1* and Ad.*BMP2* (Fig 8A). High rates of cell proliferation were observed in all groups with highest values for the Ad.*BMP2 transduced* group without major differences between the other groups (Fig 8A). The same pattern was seen in the DNA assay, where high values were observed in all groups without major differences between the Ad.*GFP* transduced control and the other groups (Fig 8B).

The extracellular matrix produced by chondrocytes in hyaline cartilage contains vast amounts of GAGs. Therefore, the GAG assay was performed to compare quantitative synthesis of extracellular matrix components characteristic for hyaline cartilage among the differently treated groups of MSCs in type I collagen hydrogels (Fig 8C). The hydrogels containing MSCs transduced with Ad.*SOX9*, Ad.*TGFB1* and Ad.*BMP2* showed significantly elevated levels of GAG synthesis in comparison to the MSCs from the non-chondrogenic Ad.*GFP* control group (Fig 8C).

For evaluation of chondrogenic hypertrophy we analyzed ALP activity within the different hydrogels. This was found to be markedly increased at time points 7, 14 and 21 in the Ad.*BMP2* transduced group and at day 21 in the Ad.*TGFB1* transduced MSC-hydrogels with no increase in the Ad.*GFP* and Ad.*SOX9* transduced MSC-hydrogels (Fig 8D).

### RT-qPCR analyzes of chondrogenic and hypertrophy marker gene expression

**Fig 9:**
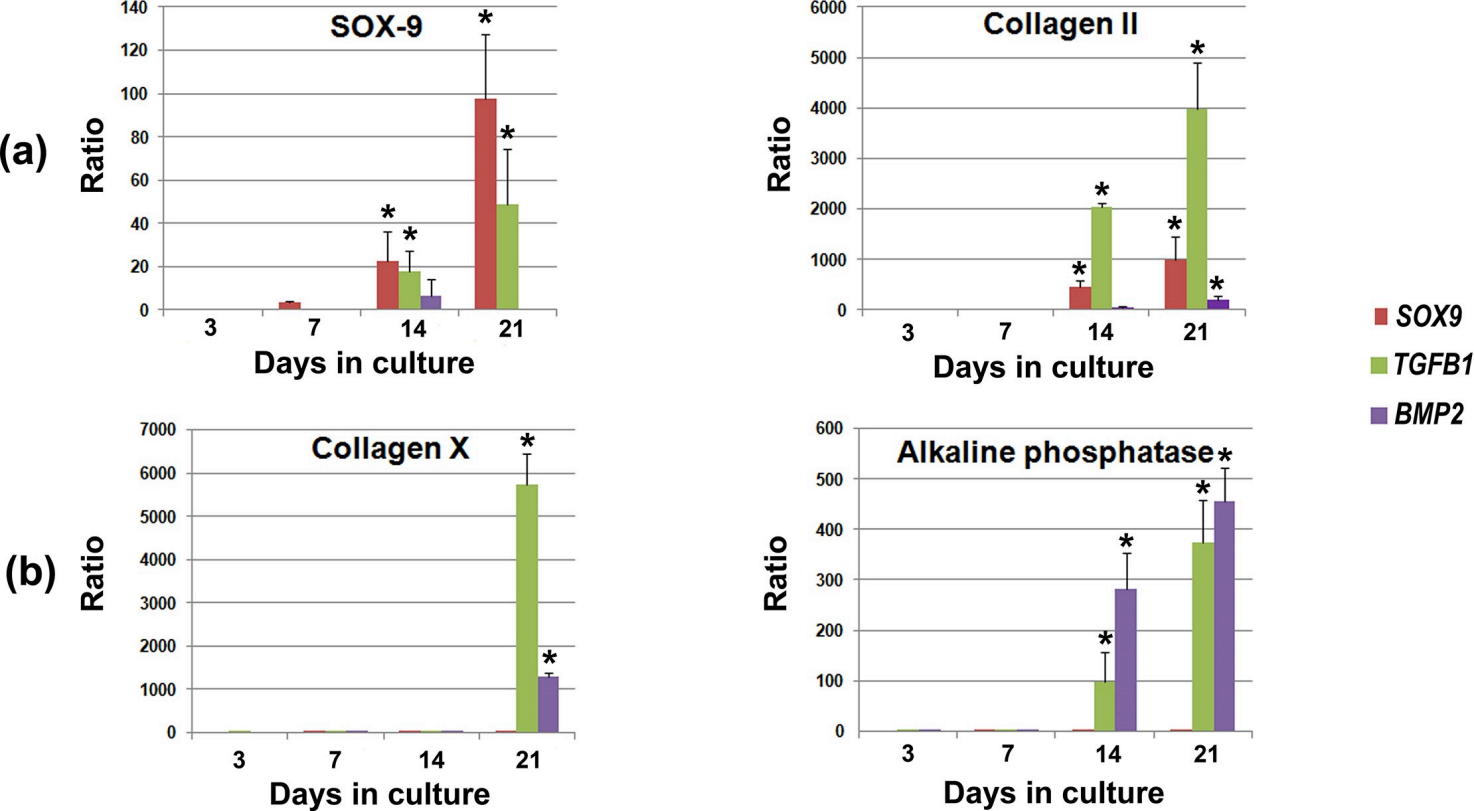
Gene expression profiles determined by quantitative real-time PCR in MSC hydrogels after adenoviral gene transfer of *SOX9*, *TGFB1* or *BMP2*. The chondrogenic marker genes studied include type II collagen alpha 1 (*COL2A1*) and *SOX9* (panels a), whereas the hypertrophic markers include type X collagen alpha 1 (*COL10A1*) and *ALPL* (panels b). Primer sequences and product sizes are presented in Table 2, with elongation factor α (encoded by *EFAA1*) serving as housekeeping gene and internal control. RNA was extracted from 3 hydrogels for each preparation/patient, treatment group and time point, and n = 3 were analysed depending on group and time point. For quantitative RT-PCR analysis values are mean +/- SD and normalized using the *EFAA1* reaction products and the ΔΔ-C_T_ method. Asterisks indicate statistically different values (p < 0.05) from the *GFP*-transduced control group.

The temporal expression profiles of chondrogenic and osteogenic differentiation markers following gene delivery of the factors *GFP*, *SOX9*, *TGFB1* and *BMP2* were analyzed by quantitative real-time RT-PCR (Fig 9, Table 2). For the assessment of chondrogenic maturation the marker genes *SOX9* and COL II alpha 1 (*COL2A1*) were included, whereas possible hypertrophic differentiation was examined using the marker genes COL X alpha 1 (*COL10A1)* and *ALPL*.

At days 14 and 21 *SOX9* and *COL2A1* were significantly upregulated in the transgene groups *SOX9*, *TGFB1* and *BMP2* compared to the negative control group *GFP* (Fig 9). Evidence of chondrocyte hypertrophy, signaled by an increase in *COL10A1* mRNA expression, was seen in MSC-hydrogels modified with *TGFB1* or *BMP2* at day 21 (Fig 9). Furthermore we found an up-regulation of the hypertrophic marker gene *ALPL* at days 14 and 21 in the Ad.*TGFB1* and Ad.*BMP2* transduced MSCs (Fig 9). In contrast, these hypertrophic mRNAs were very low or even undetectable in the Ad.*GFP* and Ad.*SOX9* transduced MSCs contained in type I collagen hydrogels.

## Discussion

The triad of CTE consists of cells, whose differentiation is initiated and maintained by specific growth factors and which are delivered to their target site through adequate biomaterials [35]. In recent years all three contributing factors have been subject to extensive research, revealing a series of obstacles which have to be overcome in order to enable a general clinical use in cartilage regeneration [2, 35]. Among these hurdles are chondrogenic hypertrophy, the search for effective delivery methods of chondrogenic growth factors *in vivo* as well as for biomaterials with potential use for *in vivo* application [2, 36].

We and other researchers previously examined the use of gene therapy as a possibility to enable an autonomous, long-lasting production of chondrogenic growth factors by MSCs [14, 16, 30]. The single or combined adenoviral gene transfer of *TGFB1* and *BMP2* promoted chondrogenic differentiation in MSCs when using pellet culture [15]. Later we and others found that the gene transfer of *SOX9* not only led to chondrogenic differentiation in MSCs but also reduced chondrogenic hypertrophy in comparison to delivery of *TGFB1* or *BMP2* [23, 30].

In our present *in vitro* study we transferred our earlier findings from pellet culture to the use of type I collagen hydrogels which have shown great potential for in vivo application in CTE [9, 12, 13]. Histological, immunohistochemical and molecularbiological results showed that adenoviral transfer of cDNAs encoding Ad.*TGFB1*, Ad.*BMP2* or Ad.*SOX9* led to successful chondrogenic differentiation in MSCs after three weeks of culture in type I collagen hydrogels. In comparison to the transfer of *SOX9* and *BMP2*, the transduction with *TGFB1* led to a significant higher expression of *COL2A1*. The gene transfer of *BMP2* more than *TGFB1* also induced a significant upregulation of the hypertrophy marker gene ALP and positive immunostainings for COL X and ALP. While adenoviral transfer of *SOX9* still led to clear chondrogenesis in MSCs, levels of chondrogenic hypertrophy after 21 days of culture in type I collagen hydrogels were significantly lower as shown by ALP activity, immunostainings and qPCR.

Hence, adenoviral transfer of Ad.*TGFB1*, Ad.*BMP2* or Ad.*SOX9* led to chondrogenic differentiation but also different levels of chondrogenic hypertrophy in MSCs. This proved that our earlier findings regarding the different effects of gene transfer of *TGFB1* and *SOX9* to MSCs could not only be observed when using pellet cultures but also when culturing MSCs in type I collagen hydrogels which are already in clinical use for CTE [30]. This technology may be used to overcome the search for effective delivery methods of cells and chondrogenic growth factors *in vivo* while offering possibilities to regenerate specific zones of native cartilage upon damage.

Earlier Nöth et al. showed that the combination of type I collagen hydrogels with soluble growth factors such as rhTGF-β1 and rhBMP-2 successfully induced and maintained chondrogenesis in MSCs *in vitro* [13]. When comparing the effects of TGF-β1 with BMP-2, the synthesis of cartilage-specific matrix proteins and the upregulation of chondrogenic marker genes appeared to set in earlier and to be stronger in TGF-β1 treated cultures. Interestingly, both groups also showed signs of hypertrophy after 21 days, with the upregulation of marker genes and proteins specific for hypertrophy setting in earlier in cultures treated with TGF-β1 which is in line with our data [13]. Jiang et al. showed that genetic silencing of *SOX9* prevented chondrogenic differentiation in MSCs when cultured in type I collagen hydrogels in a medium containing TGF-β1 [37]. In contrast, gene transfer of *SOX9* to MSCs led to improved chondrogenic differentiation *in vitro* while suppressing hypertrophy and osteogenic differentiation when cultured in pellet-aggregates or fibrin-polyurethane scaffolds [23, 24]. This is in accordance to our findings in which adenoviral gene transfer of *SOX9* to MSCs led to clear chondrogenesis in MSCs when cultured in type I collagen hydrogels for 21 days. In contrast to our findings Kupcsik et al. only observed GAG synthesis in MSCs cultured in hydrogels only when combining gene transfer of *SOX9* with mechanical stimulation [38].

We used type I collagen hydrogels to examine the effects of gene transfer on chondrogenesis in MSCs in combination with clinically approved biomaterials [10, 11]. Natural scaffolds, like the type I collagen hydrogel, are attractive for their great biocompatibility and biodegradability through natural enzymes such as collagenase, which would ideally occur at the same speed as neo-cartilage is rebuilt [36]. In addition, scaffolds formed by collagen interact with seeded MSCs and local cells promoting cellular interactions, cellular signaling and production of cartilage-specific matrix proteins [9]. Besides promoting the synthesis of a hyaline cartilage-specific ECM these natural materials have also been shown to maintain the phenotype of chondrocytes *in vitro* [39, 40]. Because hydrogels are highly hydrated, they partly reproduce the natural environment of the ECM in hyaline cartilage [6]. Interestingly, type II collagen scaffolds blended with chondroitin led to a greater deposition of cartilage-specific matrix proteins and preservation of chondrogenic phenotype than type I collagen scaffolds seeded with chondrocytes [9]. Despite these findings one major disadvantage counteracting the benefits of natural type II collagen hydrogels are possibly pro-arthritogenic effects *in vivo* that significantly restrict their possible clinical use, which is why we used type I collagen hydrogels in this study [41].

The combination of natural or synthetic biomaterials with gene transfer of chondrogenic growth factors to MSCs has already produced promising results in a number of animal *in vivo* studies [42]. The adenoviral transfer of *SOX9* to MSCs which were seeded in polyglycolic acid scaffolds significantly improved the repair of full-thickness cartilage defects in rabbits [22]. Further the transduction of MSCs with *TGFB1* led to improved repair of full-thickness cartilage defects in rats when seeded in polyglycolic acid scaffolds [43].

When regarding the future use of biomaterials for CTE the additional or single use of other components such as biotechnological chondroitin or hyaluronic acid could provide different benefits for the chondrogenic differentiation of MSCs while synthetic polymers allow modifications of structural and surface conditions [42, 44, 45]. In addition, bi- or multiphasic scaffolds may allow a better simulation of the osteochondral interface and the complex structure of hyaline cartilage to further improve the formation of different stages of chondrogenesis in seeded cells [46, 47]. Finally, safe ways of delivering the factors we investigated are necessary that meet regulatory guidelines, before such promising approaches can be advanced to clinical application.

## Conclusion

The combination of adenoviral gene transfer of either *TGFB1*, *BMP2* or *SOX9* to MSCs with *in vitro* culture in type I collagen hydrogel constructs led to successful chondrogenesis after 21 days. Our data suggests that *TGFB1* and *BMP2* rather than *SOX9* induced a significant hypertrophic response in bone-marrow MSCs seeded in type I collagen hydrogels, resulting in different types of neo-cartilaginous tissues. This technology might be used to regenerate the zonal architecture of hyaline cartilage *in vivo*. However, to further examine and optimize the effect of gene transfer of *SOX9*, *TGFB1* or *BMP2* to MSCs for CTE, safe ways of delivery have to be explored in conjunction with confirmation in meaningful *in vivo* studies, before this research can be advanced to clinical application.

## Supporting information

S1 Fig SOX9-Transgen. (JPG)

S2 Fig GFP-Transgen. (JPG)

S1 Data qRT-PCR. (XLSX)

S2 Data TGFb1-ELISA. (XLSX)

S3 Data BMP2-ELISA. (XLSX)

S4 Data ALP-Assay. (XLSX)

S5 Data DNA-Assay. (XLSX)

S6 Data GAG-Assay. (XLSX)

S7 Data ATP-Assay. (XLSX)
