# Supplementary figures and images for: Different types of cartilage neotissue fabricated from collagen hydrogels and mesenchymal stromal cells via SOX9, TGFB1 or BMP2 gene transfer
Source: PLoS One. 2020 Aug 13;15(8):e0237479. doi: 10.1371/journal.pone.0237479 (PMC7425924; doi:10.1371/journal.pone.0237479)

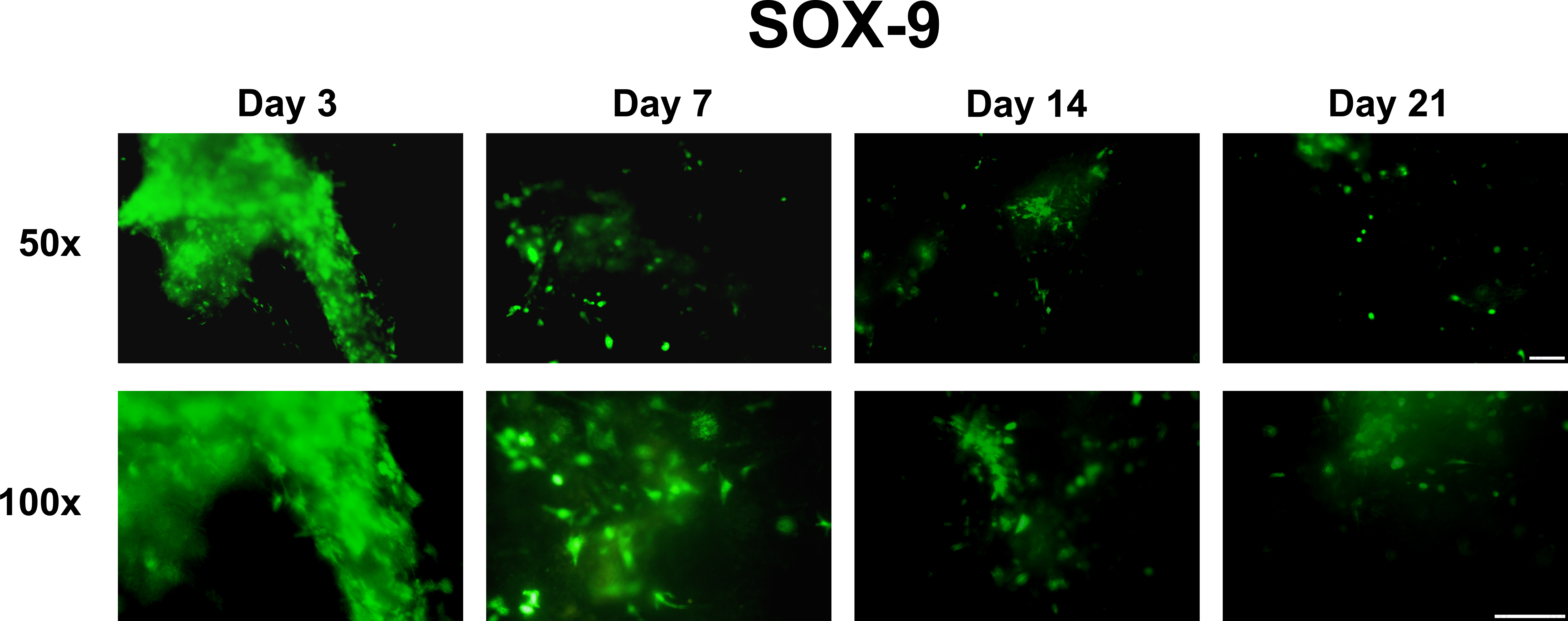

Supplement: S1 Fig — (JPG) [file pone.0237479.s001.jpg]

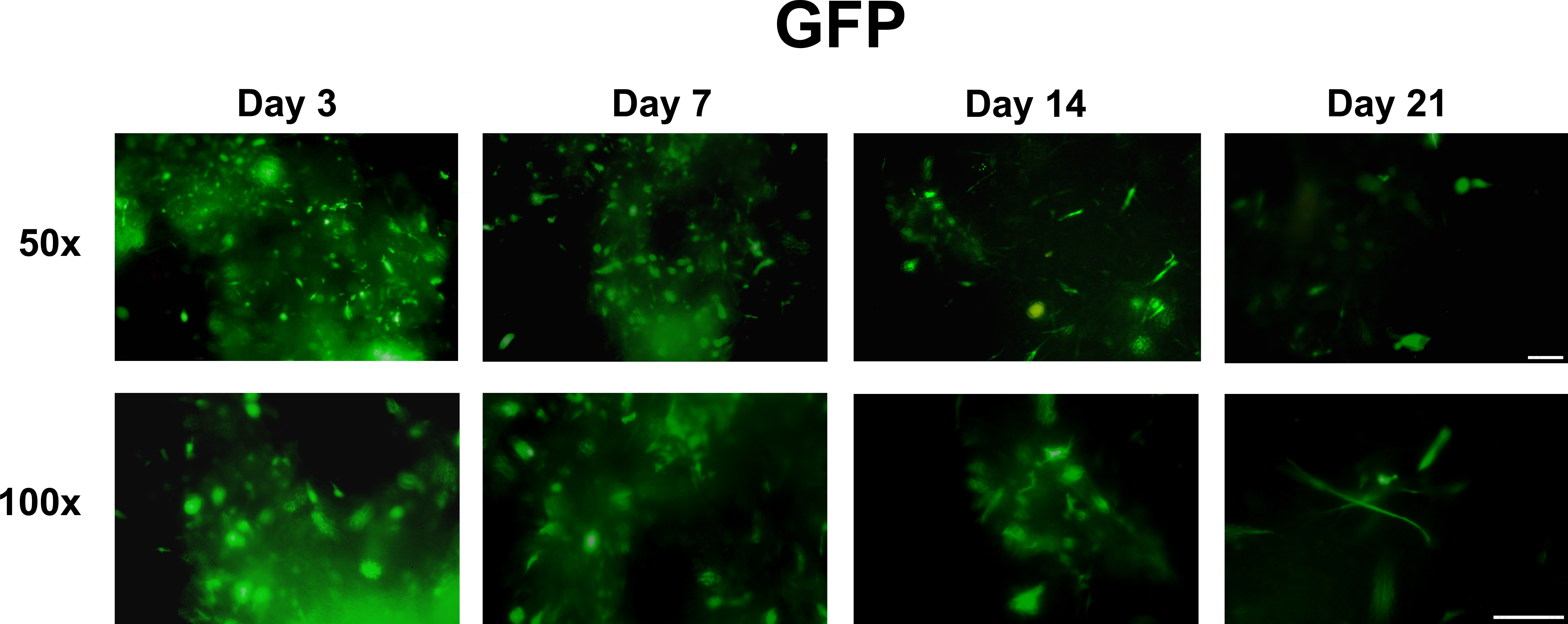

Supplement: S2 Fig — (JPG) [file pone.0237479.s002.jpg]
